# Route of pesticide spread on the body surface of *Blattella germanica* (Linnaeus): a NanoSuit–energy dispersive X-ray spectroscopy analysis

**DOI:** 10.1038/s41598-023-41474-x

**Published:** 2023-08-31

**Authors:** Yasuharu Takaku, Katsumi Shiraki, Chiaki Suzuki, Sayuri Takehara, Hiroyuki Nishii, Tomonori Sasaki, Takahiko Hariyama

**Affiliations:** 1 Laboratory of Bio-Design, Department of Agricultural Innovation for Sustainable Society, Tokyo University of Agriculture, 1737 Funako, Atsugi, Kanagawa 243-0034 Japan; 2 NanoSuit Inc., 1-20-1 Handayama, Higashi-ku, Hamamatsu, 431-3192 Japan; 3 Research and Development Division, Fumakilla Limited, Umehara 1-11-13, Hatsukaichi, Hiroshima 739-0494 Japan; 4 Preeminent Medical Photonics Education & Research Center, Institute for NanoSuit Research, Hamamatsu University School of Medicine, 1-20-1 Handayama, Higashi-ku, Hamamatsu, Shizuoka 431-3192 Japan

**Keywords:** Scanning electron microscopy, Agroecology

## Abstract

Numerous studies have focussed on the mechanisms of entry of pesticides into insect body parts such as oral intake, penetration through the integument of the body wall, and inhalation through spiracles. However, little is known about how insecticides spread to the points of entry or the paths on the body surface that are used to reach the target sites. In this study, elemental signals of pesticide-mimicking test solutions were tracked and their routes of spreading in experimental insects (*Blattella germanica* L.) were investigated using NanoSuit (a method of surface modification) and energy dispersive X-ray spectroscopy, combined with high-resolution scanning electron microscopy. When the test solution initially adhered to the dorsal and/or ventral body surface, it tended to spread horizontally to reach lateral plates. Whereas, when the solution directly adhered to the anterior side of the lateral plates, it spread to posterior segments. In this case, however, spreading in the opposite direction (i.e., the solution directly adhered to the posterior side of the lateral plates) was interrupted at a boundary erected by different groups of fine structures; each protrusion was large, and the arrangement was rather dense in the posterior segments. Morphological features of these fine structures and chemical characteristics of the hydrophobic surface substances potentially regulate the strength of the capillary force, which determines pesticide spreading.

## Introduction

Pesticides have become indispensable for increasing the food production. Insect pest extermination has been extensively researched since the last 200 years^1^. Initially, it was believed that the more pests were exterminated, the more crops could be harvested^2^. However, only approximately 0.1% of the applied pesticides affect target organisms, and non-target organisms inadvertently face most of the adverse effects of pesticides^2,3^. Moreover, even though pesticides eliminate the target species from a certain area, other pests eventually emerge in such environments to exploit the newly unoccupied niches^4^.

Despite being considered as pests, some insects provide important ecological services such as visiting flowers/pollination, herbivory and detrivory, and serving as food sources for other organisms^5–9^. Thereby, the loss of insect diversity and abundance would lead to chain reactions in food webs and hamper the provision of ecosystem services. Accordingly, in recent years, reports of declining insect populations on a global scale have raised widespread concern, with insect pest control being associated with economic and ecological challenges^9–11^. Owing to conflicting human demands between higher food production and proper ecological conservation, it is recommended that pesticides must possess properties such as high specificity, high insecticidal activity, and relatively low toxicity to other creatures such as animals and plants.

Various studies have addressed the entry routes of specific pesticides into the insect body through oral intake, penetration through the integument of the body wall, and inhalation through spiracles. Lovell reported that the effective barrier capacity of the integument not only varies between different insect species but also from one insecticide to another^12^. Gerolt suggested that the integument of the tracheal system is the most important site for rapid knockdown^13^. Alternatively, upon investigating the behaviour of topically applied pyrethroids, Sugiura et al. found that the effective entry for rapid knockdown resulted from the flow of the insecticide into mesothoracic spiracles along the lateral plates in German cockroaches^14^. Despite many investigations, little is known about the mechanisms behind spreading of insecticides into points of entry or paths on the body surface of insects to reach target sites. By elucidating the spreading routes of insecticides, we will have some chances to select and develop appropriate and sustainable pesticides for target insects, and minimise the amount of application and collateral damage.

Scanning electron microscopy (SEM) facilitates the observation of nano-sized fine structures of biological specimens at a high resolution^15^. In a previous study, we developed a method for keeping select insects alive in the high vacuum environment of an electron microscope by encasing them in a thin, vacuum-proofed suit, termed the NanoSuit^16–18^. Additionally, we described the specific properties of the NanoSuit and examined its application in the elemental analysis of biological specimens, using energy dispersive X-ray spectroscopy (EDS) performed with SEM^19,20^. This method facilitates the accurate detection of in situ elemental compositions on living specimens at a high resolution. In the present study, to examine the relationship between the insects and the real pesticide, we use *Blattella germanica* L. (German cockroaches) larvae as the experimental specimen and a pesticide-mimicking hydrophobic solution. By following the elemental signals of the test solutions, we investigated the spread of pesticides on body surfaces protected with a NanoSuit under a high-resolution SEM equipped with an EDS system (cf. Supplementary Movie 1). Furthermore, we demonstrate the main routes and directions of the spreading of the hydrophobic insecticide and provide evidence to show that the spreading of liquids is based on the open capillarity in living insect specimens.

## Results

### Pesticides applied on dorsal and/or ventral body surfaces spread to lateral plates

**Figure 1:**
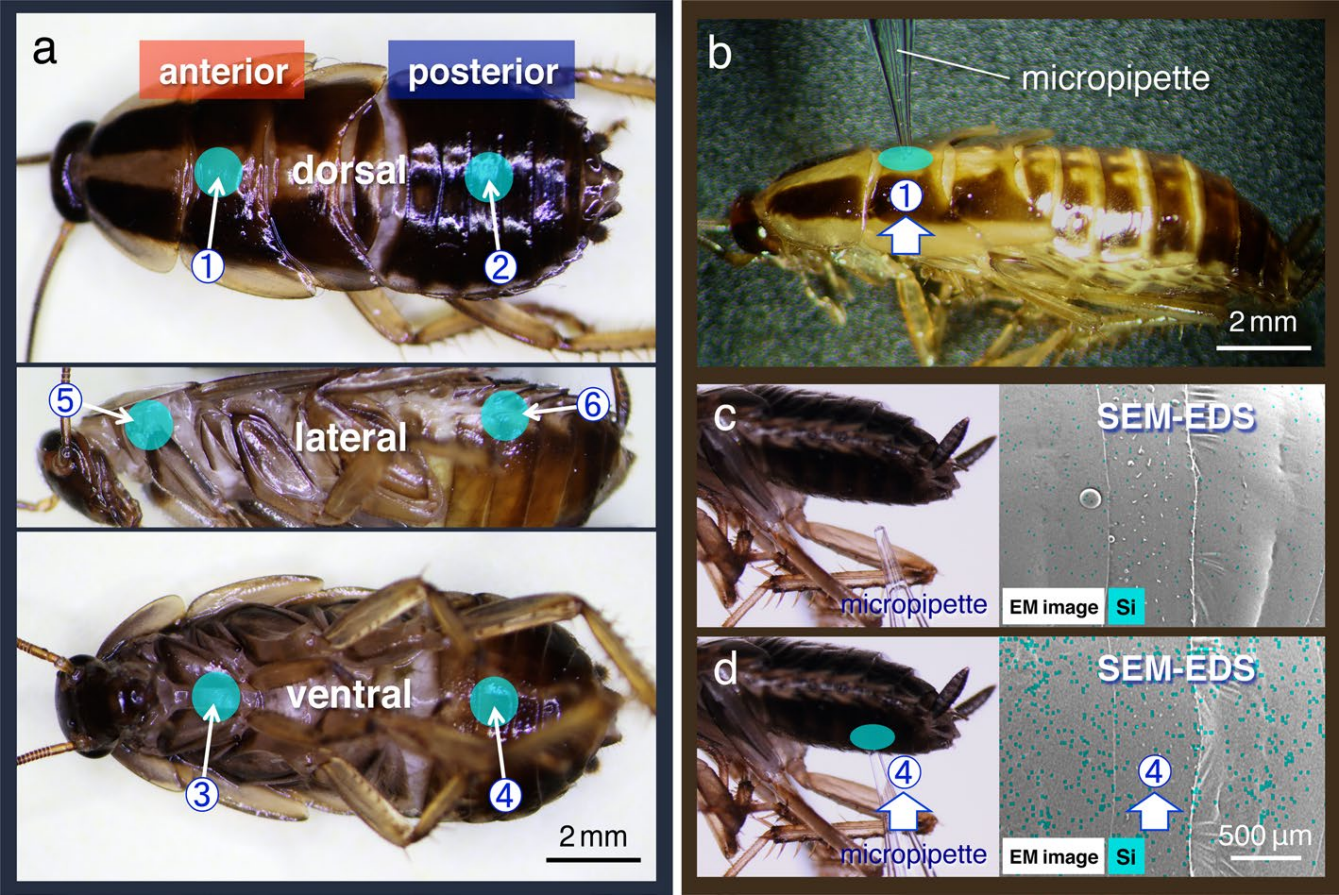
Overview of experiments involving pesticide-mimicking solutions applied to six sites on *B. germanica* specimens. (**a**) Middle of the dorsal (①, application site no. 1) or ventral surface (③, application site no. 3) in anterior body segments; middle of the dorsal (②, application site no. 2) or ventral surface (④, application site no. 4) in posterior segments; in the anterior (⑤, application site no. 5) or posterior segments (⑥, application site no. 6) in the lateral plates. (**b**) Light microscope (LM) image of adding the solution at application site no. 1 with a glass micropipette. (**c**) and (**d**) LM and scanning electron microscopy (SEM) images of energy dispersive X-ray spectrometry (EDS) mapping analysis of application site no. 4, before (in **c**) and after (in **d**) adding the solution, with the elemental silica mapping signal (Si: light blue).

**Figure 2:**
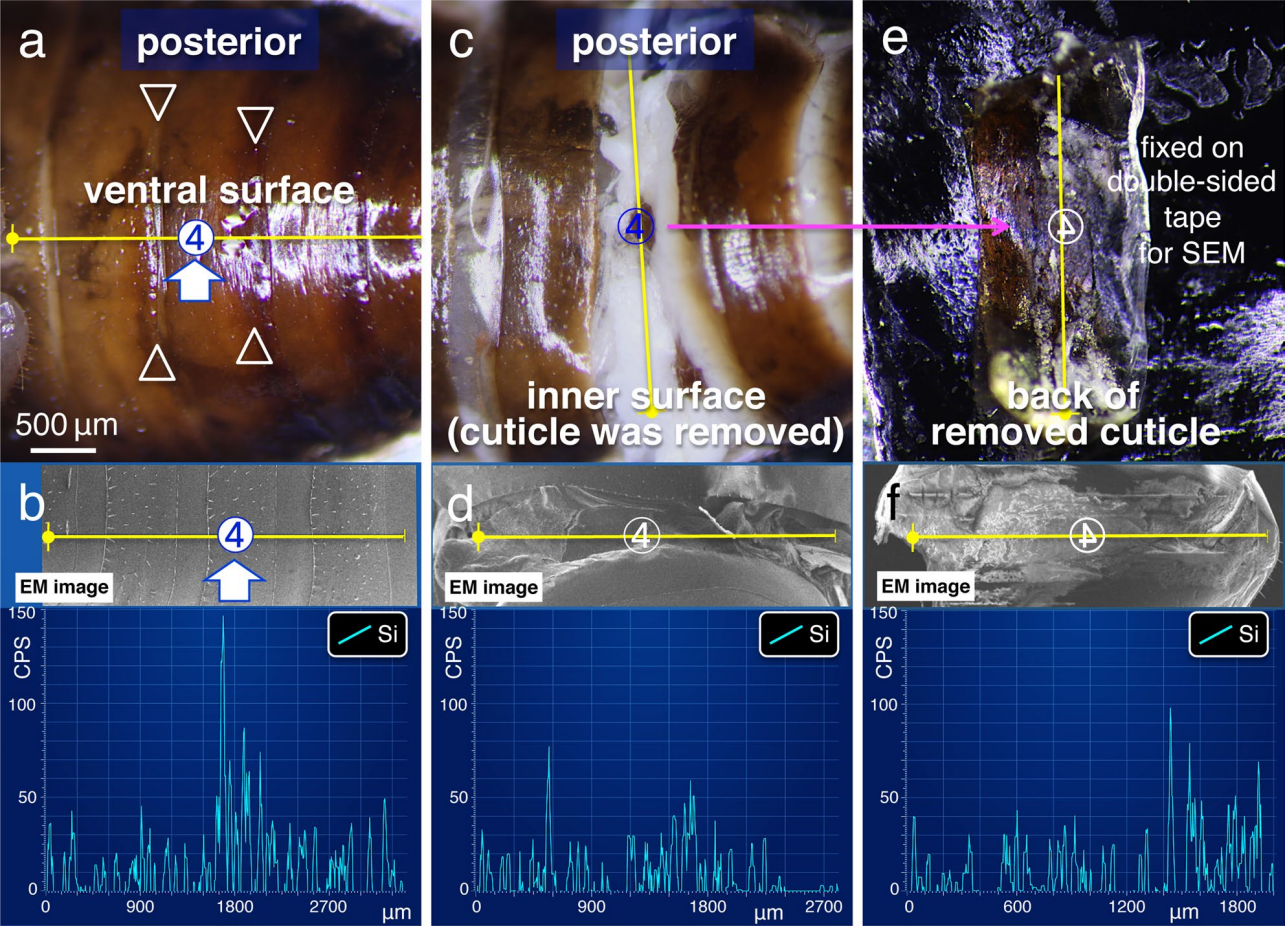
EDS “line scanning analysis” images for the quantification of spreading on outer and/or inner surfaces. (**a**), (**c**), (**e**) and (**b**), (**d**), (**f**) represent LM and the EDS “line scanning analysis” (along yellow lines) of the observation fields (shown as insets), respectively. The ventral surface in the posterior segment of application site no. 4 in (**a**) and (**b**), the inner surface where the outer cuticle was removed in (**c**) and (**d**), and the back of the removed cuticle in (**e**) and (**f**) are shown. Inverted number 4 in (**e**) and (**f**) indicates the ventral side.

Initially, the pesticide-mimicking solution was applied to four independent sites on the surfaces of specimens (*B. germanica* L.) as follows: middle of dorsal or ventral surfaces in anterior body segments (Fig. 1a, application site nos. 1 and 3, respectively); middle of dorsal or ventral surfaces in posterior segments (Fig. 1a, application site nos. 2 and 4, respectively). To elucidate the spreading mechanisms, a small volume of 0.2 μL of the pesticide-mimicking solution was used (Supplementary Fig. S1). Observations at low magnification (30–50×) under a stereo dissecting microscope showed that following application on to the body surface, test solutions spread quickly along the arthrodial membranes that run perpendicular to the anterior–posterior body axis (Supplementary Movie 2) and moved to the lateral plate. Owing to capillary forces in the valleys of arthrodial membranes, we found that spreading was not associated with gravity. Therefore, the solution moved in the same direction even when it was added from below (Fig. 1b–d). To quantify spreading, we performed EDS analyses of specimens protected with the NanoSuit^20^. Since the test solution in the present study contained silicon oil, silica could be detected at locations where the solution had passed (See Methods). Analysis using a field-emission SEM (FE-SEM) revealed that silica peaks were higher at positions where the test solution was applied (Fig. 2a,b; 93.9 ± 43.0 counter per second [CPS]; mean ± standard deviation (SD) added at the application site no. 4, *N* = 10; Table 1), whereas the values were lower at positions along the anterior–posterior body axis. Once the solution spread, the localisations of silica did not change over time during the SEM–EDS analysis.

**Table 1:** Relationship between application site and silica element (counter per second [CPS]) determined using EDS. A topical application of 0.2 μL of the pesticide-mimicking test solution was used (*N* = 10).

Application site	Surface of cuticle	Lateral plate
①	77.2 ± 29.8	518.7 ± 184.4
②	91.9 ± 23.3	464.9 ± 133.8
③	101.7 ± 34.1	445.7 ± 164.1
④	93.9 ± 43.0	419.7 ± 152.4
(④)	35.6 ± 21.2 (inner surface)	ND
(④)	41.5 ± 30.7 (back of cuticle)	ND

To examine whether the solution had entered body tissues, we gently removed the cuticle covering the external surface of the specimens. Several silica peaks were observed on the surfaces of inner tissues (Fig. 2c,d; 35.6 ± 21.2 CPS; mean ± SD, *N* = 10; Table 1) and on the back of the removed cuticle (Fig. 2e,f; 41.5 ± 30.7 CPS; mean ± SD, *N* = 10; Table 1). These results suggest that although only a small quantity of solution penetrated the body, the spreading mainly occurred horizontal to the body axis on the dorsal and/or the ventral body surface.

### Directional movements in lateral plates via open capillarity: role of dense short protrusions

**Figure 3:**
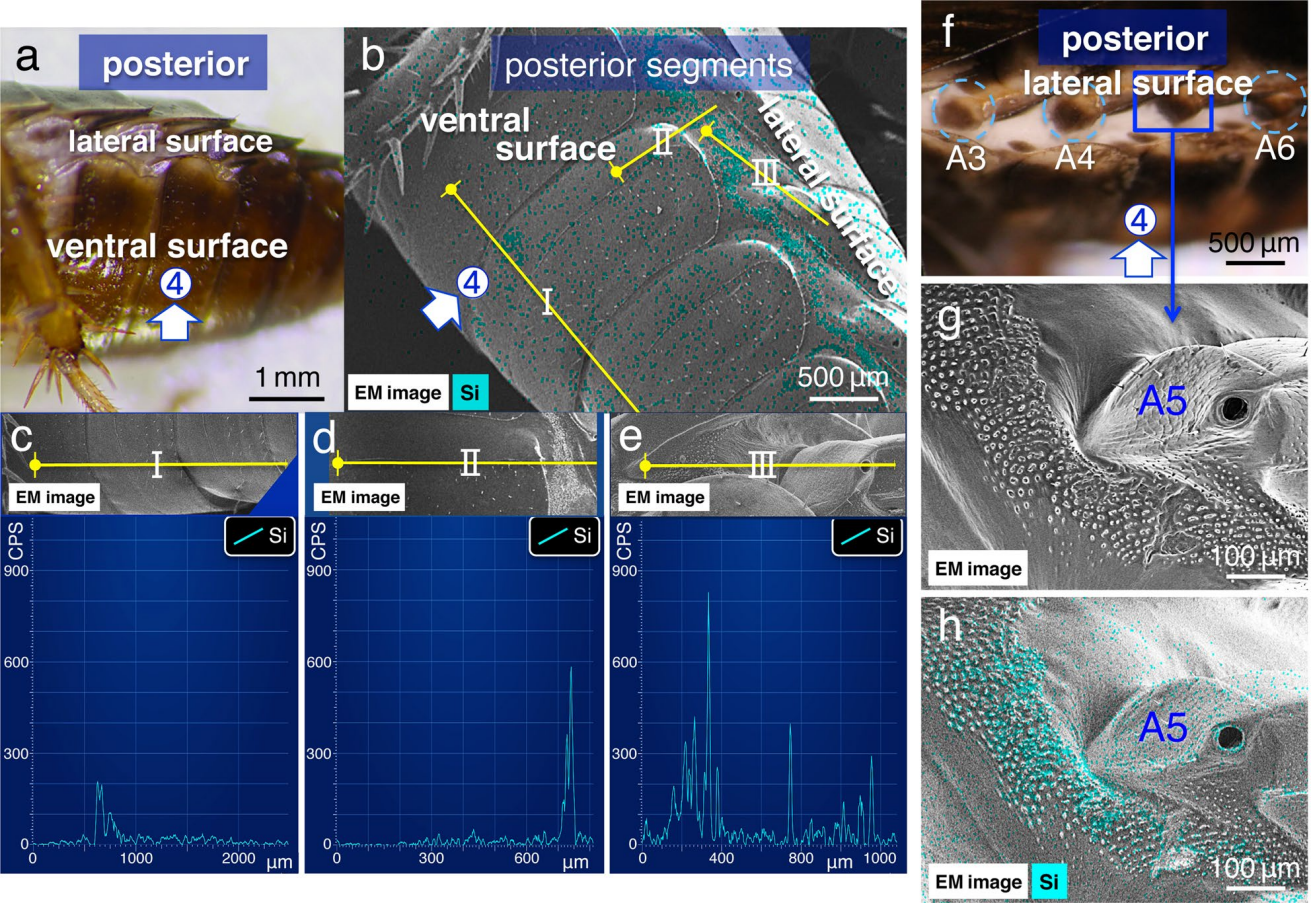
Images of the EDS analyses: spreading from the ventral body surface to lateral plates. (**a**) Light microscope (LM). (**b**–**e**) Images from the EDS “mapping analysis” near application site no. 4 in (**b)**, and EDS “line scanning analysis” (along yellow lines) of the same observation field (shown as insets) in (**c**–**e**). I–III indicate the positions analysed using EDS in (**b**). (**f**) LM. (**g**) Electron microscope (EM). (**h**) EDS “mapping analysis” images of characteristic fine structures with short protrusions in the lateral plate. Light blue signal indicates the localisations of elemental silica. A3–A6: abdominal spiracles (cf. Fig. 6a).

Subsequently, we examined silica signals on and around the lateral plates in the unfixed living specimens and found high peaks in these regions (Fig. 3a–e). Regardless of the test solution at the four application sites, all values in the lateral plate were higher than those at the four initial application sites (419.7 ± 152.4 CPS; mean ± SD added at the application site no. 4, *N* = 10; *p* < 0.01; Table 1). Observations with SEM under higher magnifications (2000–5000×) revealed that silica signals were strongly localised at unique fine structures with dense and short protrusions (Fig. 3e–h; Supplementary Fig. S1) that run parallel to the body axis on the lateral plate (Fig. 3b). In addition, when the test solution was added to one ventral (or dorsal) surface, although high peaks were detected in that particular lateral plate, similar strong signals were not observed on other dorsal (or ventral) surfaces (*N* = 10, in each experiment in Table 1). This suggests that once the spreading solution reaches the lateral plate, it accumulates at the site and is unlikely to reach other body surfaces based on the volume of pesticide used.

**Figure 4:**
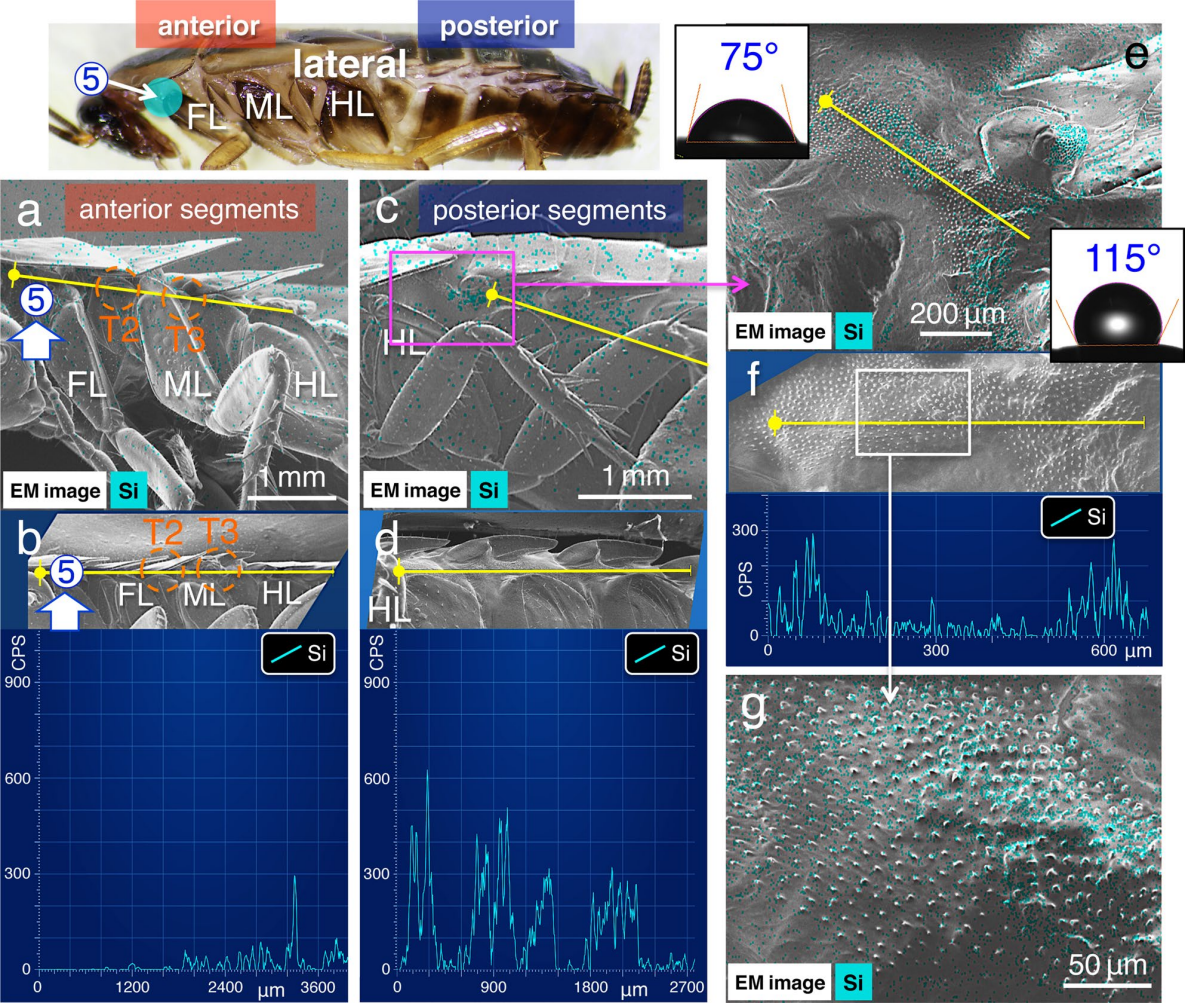
Images of EDS analyses: spreading from the anterior to posterior segments along lateral plates. (**a**) and (**c**) Images from the EDS “mapping analysis” near application site no. 5 in the anterior segments in (**a**), and in the posterior segments in (**c**), (**b**) and (**d**) EDS “line scanning analysis” (along yellow lines) of the same observation field (shown as insets). (**e**) and (**g**) Images of the EDS “mapping analysis,” and (**f**) “line scanning analysis” (along yellow lines) of the fine structures with short protrusions in the middle of the lateral plate. Water contact angle measurements are shown in the insets in (**e**). Light blue signal indicates the localisations of elemental silica. FL: front leg, ML: middle leg, and HL: hind leg. T2 and T3: mesothorax and metathorax spiracles, respectively (cf. Fig. 6a).

**Figure 5:**
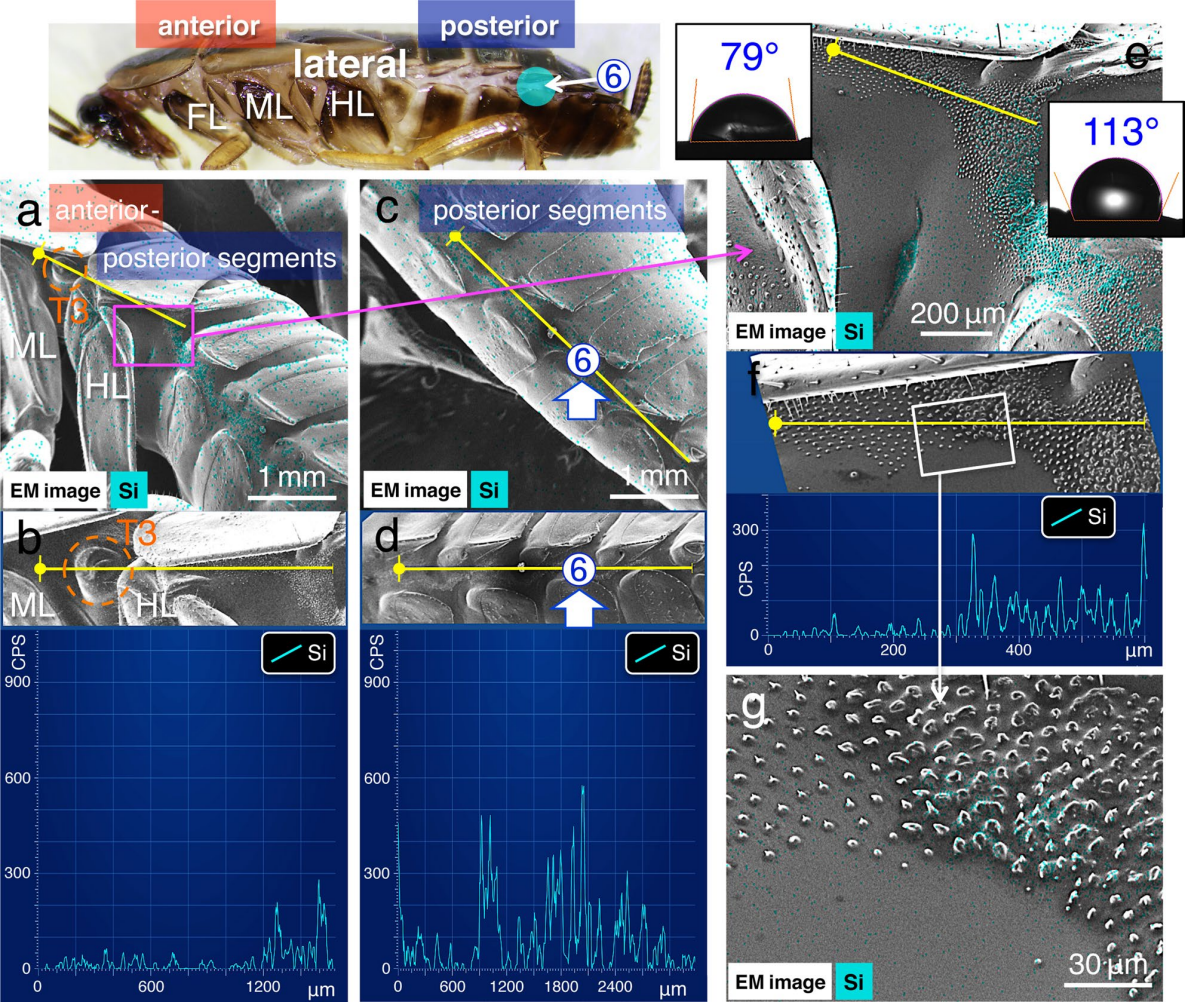
Images of EDS analyses: spreading from the posterior to anterior segments along lateral plates. (**a**) and (**c**) Images from the EDS “mapping analysis” in the anterior segments in (**a**), and near application site no. 6 in the posterior segments in (**c**). (**b**) and (**d**) EDS “line scanning analysis” (along yellow lines) of the same observation field (shown as insets). (**e**) and (**g**) Images from the EDS “mapping analysis”. (**f**) “line scanning analysis” (along yellow lines) of the fine structures with short protrusions in the middle of the lateral plate. Water contact angle measurements are shown in the insets in (**e**). Light blue signal indicates the localisation of elemental silica. Note, that the signal is disconnected at the “boundary” shown in (**g**). FL, front leg; ML, middle leg; HL, hind leg. T3: metathorax spiracle (cf. Fig. 6a).

**Figure 6:**
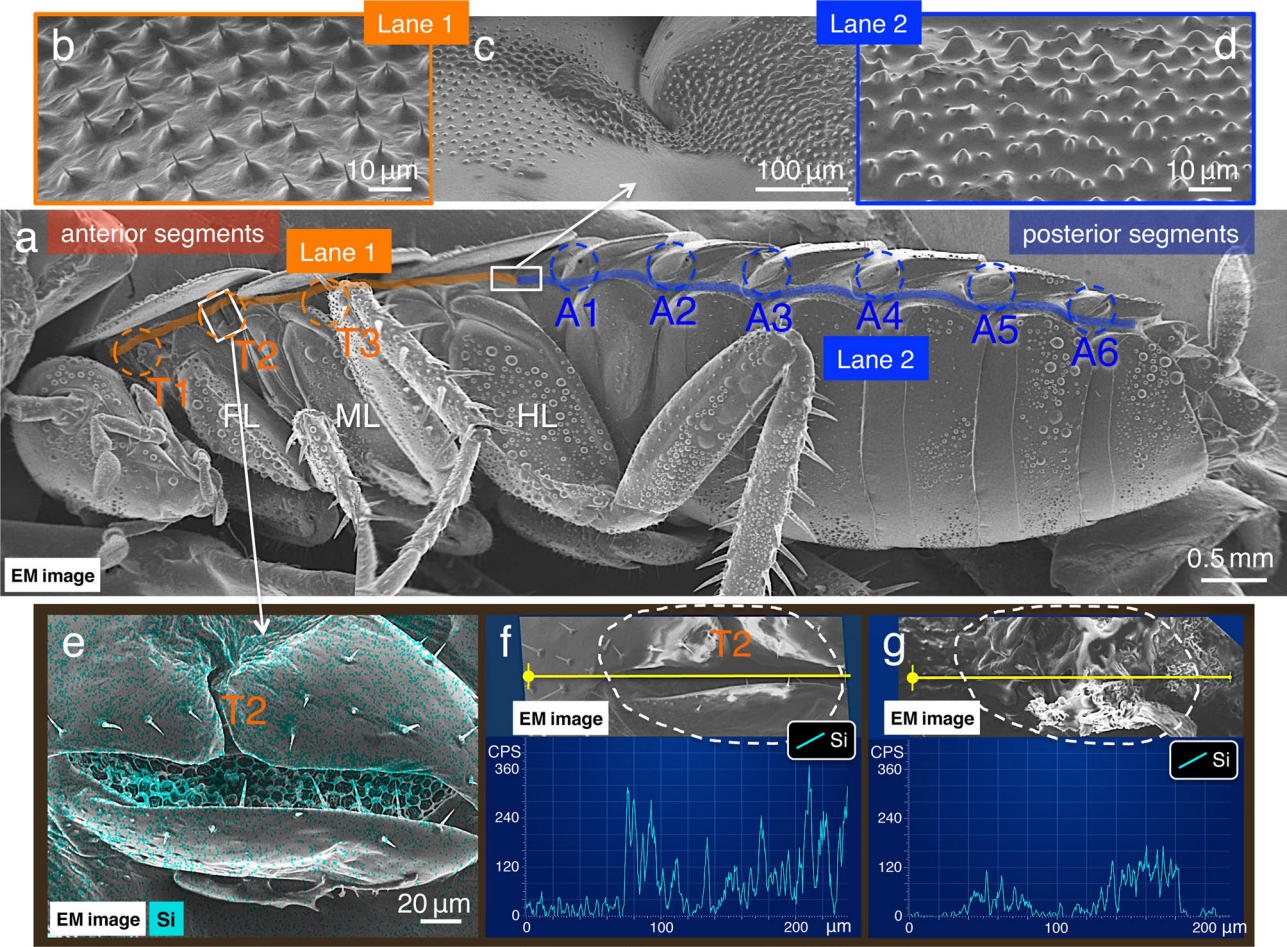
Electron microscope (EM) images of the lateral plates and the EDS analysis in the spiracles. (**a**) An EM image of the lateral plate showing the positions of prothorax (T1), mesothorax (T2), and metathorax (T3) spiracles, and the six abdominal spiracles (A1–A6). (**b**), (**c**), and (**d**) Morphological features of dense and short protrusions in anterior segments in (**b**) and (**c**) (along lane 1 in **a**), and those in the posterior segments in (**c**) and (**d**) (along lane 2 in **a**). (**e**) EDS “mapping analysis” of mesothoracic spiracles in which the test solution was applied at site no. 5. (**f**) and (**g**) EDS “line scanning analysis” (along yellow lines) of the mesothoracic spiracle in (**f**), and the inner wall of the mesothoracic trachea, from which the surface structure of the mesothoracic spiracle was removed using forceps in (**g**). The white dotted lines in (**f**) and (**g**) indicate the outlines of mesothoracic spiracles before and after dissection, respectively. Light blue signal indicates the localisation of elemental silica. FL, front leg; ML, middle leg; HL, hind leg.

To further investigate spreading properties, test solutions were applied to site no. 5 and 6, at the anterior and posterior segments in the lateral plates, respectively (Fig. 1a). Observations under low magnification using a stereo dissecting microscope showed that when the test solutions were applied at these sites, they seemed to spread along the lateral plate (Supplementary Movie 3); however, the detail was unclear. Analysis using SEM–EDS showed that the initial application of the solution to anterior segments (application site no. 5; Fig. 4) enabling the detection of silica signals on the lateral plate in both the anterior and posterior segments (Fig. 4a–d; Table 2), where the signal was strongly localised at dense and short protrusions (Fig. 4e–g). These results suggest that the test solutions spread from the anterior to posterior segments via the capillary force of dense and short protrusions^21^. Conversely, when the solution was applied to the lateral plate in posterior segments (application site no. 6; Fig. 5), significantly higher silica signals were detected in them (Table 2); however, silica signals were disconnected in between the anterior and posterior segments (Fig. 5a–d). SEM observations under a higher magnification revealed that morphological features of the short protrusions varied at the boundary where the signal was disconnected (Fig. 5e–g). A comparison of dense and short protrusions observed along the anterior segments (Fig. 6a–c in lane 1) show that each protrusion was large, and the arrangement was rather dense in the posterior segments (Fig. 6a,c,d in lane 2; *N* = 10, in each experiment in Table 1).

**Table 2:** Relationship between application site and silica element (CPS) assessed using EDS. A topical application of 0.2 μL of the pesticide-mimicking test solution was used (*N* = 10).

Application site	Anterior segments	Posterior segments
⑤ (Control)	90.9 ± 97.1	405.1 ± 212.4
⑤ (Treated with detergent)	409.9 ± 127.3	51.3 ± 21.0
⑥ (Control)	24.9 ± 23.0	438.1 ± 256.6
⑥ (Treated with detergent)	18.2 ± 19.0	445.0 ± 207.0

Application site	Mesothorax spiracle (T2)	Abdominal spiracle (A3)
⑤	107.5 ± 30.8 (surface of spiracle)	233.2 ± 103.7 (surface of spiracle)
⑤	82.2 ± 45.3 (inner surface)	83.9 ± 36.3 (inner surface)
⑥	18.2 ± 23.7 (surface of spiracle)	191.6 ± 100.9 (surface of spiracle)
⑥	9.3 ± 9.8 (inner surface)	101.2 ± 26.8 (inner surface)

These observations prompted our hypothesis that the capillary forces in the anterior or posterior segments were controlled by varying wettability in the regions with dense and short protrusions. This could be because hydrophobic oils (lipids) or non-polar organic solvents, such as waxes, are likely to have a high affinity for a hydrophobic surface. We measured wettability using a microscopic contact angle metre on the natural surface of specimens that were not treated with the NanoSuit methods to quantify its surface affinity to the hydrophobic test solution. In the anterior segments of the lateral plate, the water contact angle (*θ*) was 82.4 ± 4.1° (mean ± SD, *N* = 10), while it was higher, 114.7 ± 2.6° (mean ± S.D., *N* = 10; *p* < 0.01), along the posterior segments of the lateral plate (Figs. 4e, 5e, insets). These results suggest that different capillary forces derived from these regions affect the property in open capillarity^21^.

The silica values detected in spiracles, which are localised along the lateral plates, also support this concept (Fig. 6e; Table 2). Following the application of test solutions at site nos. 5 and 6, using the same procedures as in the experiments in Fig. 2c,e, in which we gently removed the outer surface structures of spiracles, which were then assessed using EDS. When the solution was added to site no. 5, several silica peaks were detected both on the outer and inner surfaces of the thorax and abdominal spiracles (Fig. 6e–g; Table 2). Conversely, following the application at site no. 6, signals were higher in the abdominal spiracles, suggesting that the solutions did not spread from the posterior to the anterior segments (*p* < 0.01; Table 2).

### Chemical modification plays a role in the spreading of pesticides

**Figure 7:**
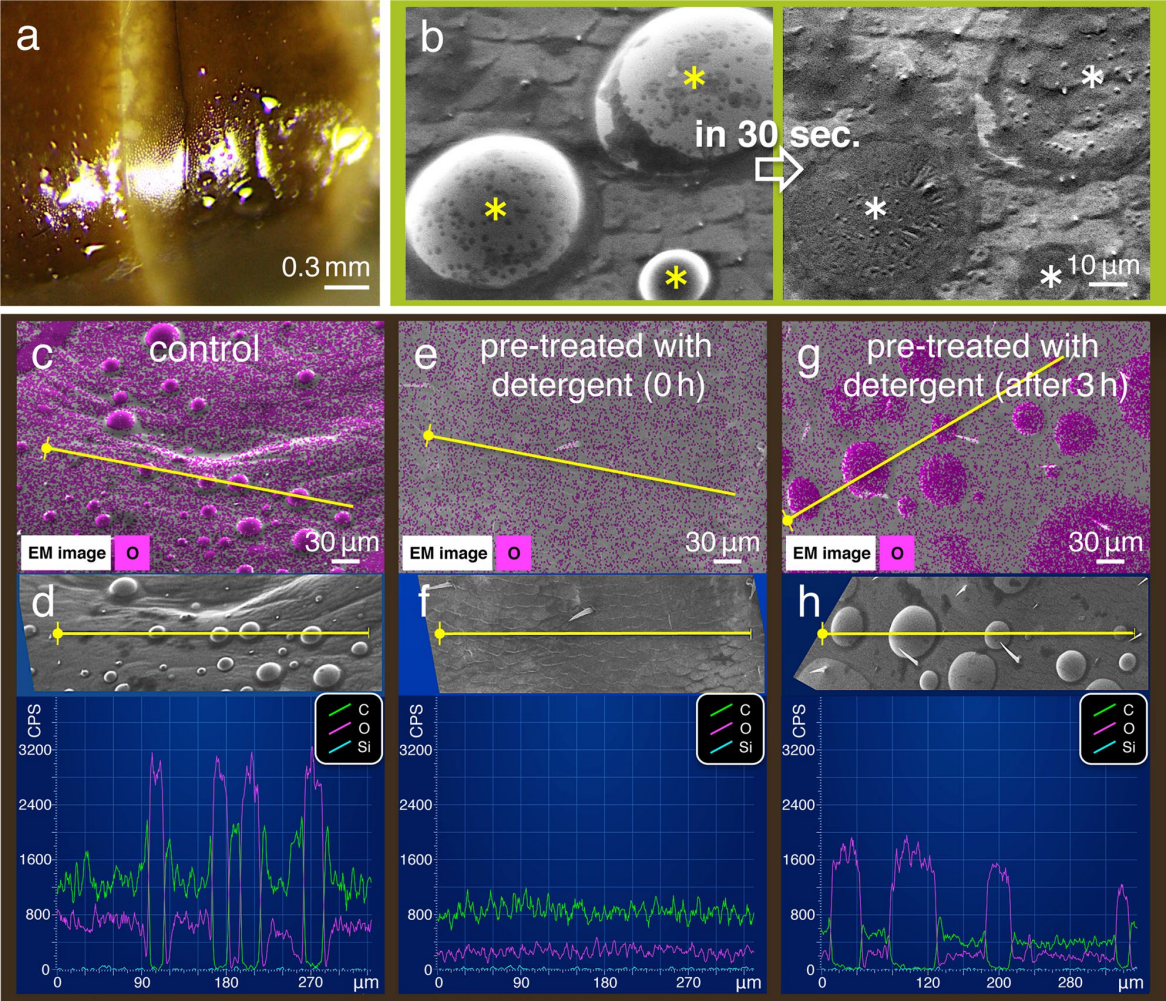
Hemispheroidal liquid substances on the surface of *B. germanica*. (**a**) Light microscope (LM) and (**b**) Electron microscope (EM) images of hemispheroidal liquid substances on the ventral surface of living *B. germanica* specimens. When the magnification increased from 1000× to 5000× in (**b**), the liquid substances evaporated within 30 s. (**c**), (**e**), and (**g**) Images from the EDS “mapping analysis”. (**d**), (**f**), and (**h**) “line scanning analysis” (along yellow lines) of these structures. Carbon (C; green), oxygen (O; magenta), and silica (Si; light blue).

We previously reported that the NanoSuit method maintains the original specific features of specimens when observed under the FE-SEM (Supplementary Movie 1)^16–18^. In the present study, unfixed living specimens were found to have another unique property; numerous hemispheroidal liquid substances were observed on the surface of cockroaches not only under atmospheric conditions under a stereo dissecting microscope (Fig. 7a) but also under high vacuum conditions under FE-SEM (Figs. 6a, 7b). These substances were not observed in specimens prepared with conventional methods of fixation. We also found that some of the liquid substances evaporated quickly when the magnification shifted from low (1000×) to high (5000×) during SEM observations (Fig. 7b; Supplementary Movie 4). These active conformational changes may be caused by the concentrated electron beam irradiating a small area using high energy. After the conformational changes occurred, we did not observe them affecting the masking ability of the NanoSuit against body fluid and water. EDS analysis further revealed that these liquid substances showed high oxygen peaks and low carbon peaks (Fig. 7c,d).

It has been reported that various organisms achieve hydrophobicity on their body surface based on physical properties of fine structures and/or chemical modifications with oils or waxes secreted on the outer surface^18,22,23^. The results presented here show that the surface of the cockroach is hydrophobic (Figs. 4e, 5e, insets) and exhibit fine structures with short protrusions and hemispheroidal liquid substances. This leads to the hypothesis that, although the solution followed specific spreading routes, spreading would be altered when the chemical property of the surface changed.

**Figure 8:**
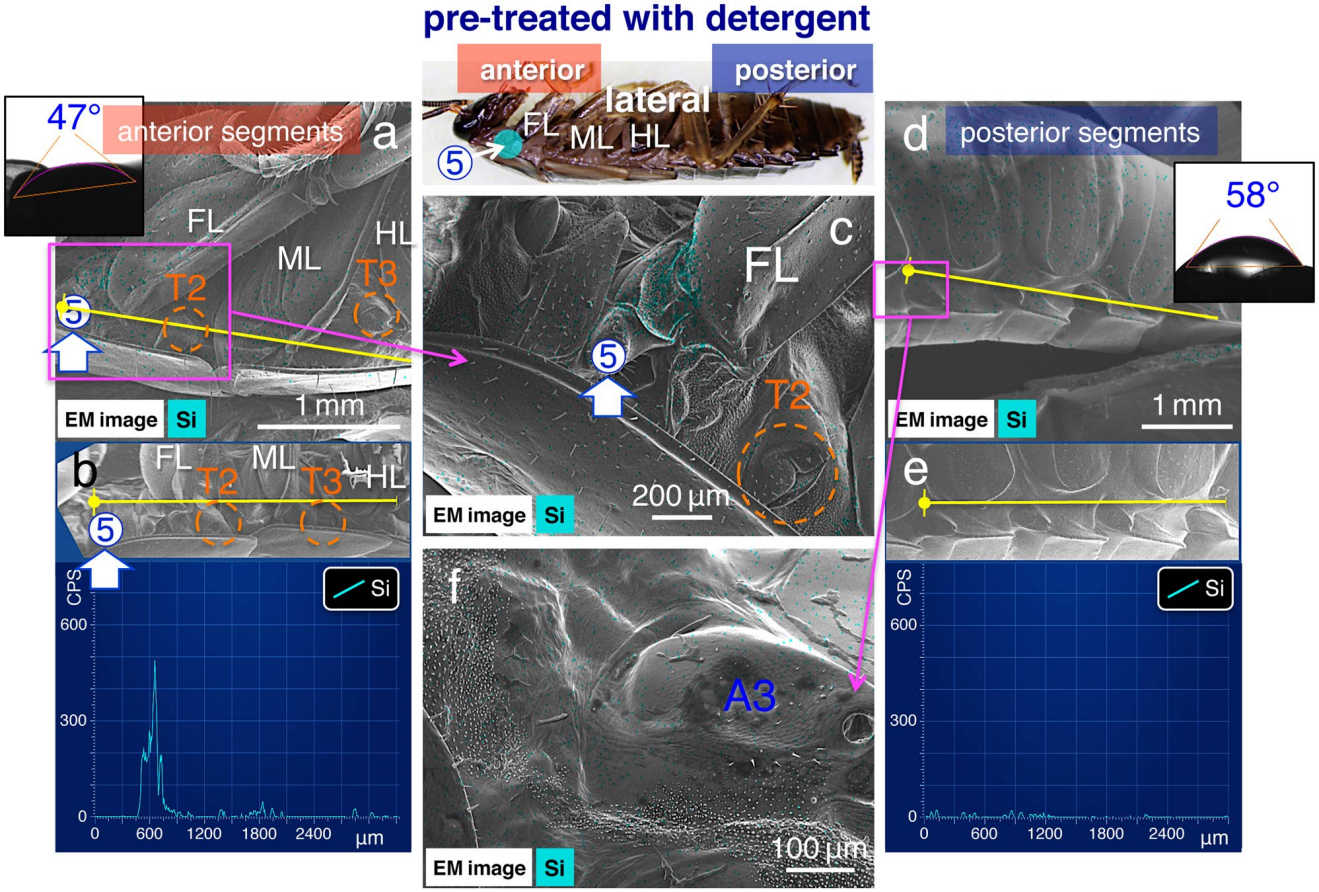
Images of the EDS analysis of specimens with reduced liquid structures. (**a**) and (**d**) Images from the EDS “mapping analysis” near application site no. 5 in the anterior and posterior segments in (**a**) and (**d**), respectively, in detergent-treated specimens. (**b**) and (**e**) EDS “line scanning analysis” (along yellow lines) of the same observation field (shown as insets). (**c**) and (**f**) High magnification images of the EDS “mapping analysis” near application site no. 5 in (**c**) and in fine structures with short protrusions in the middle of the lateral plate in (**f**). Water contact angle measurements are shown in the insets. Light blue signal indicates the localisation of elemental silica. FL, front leg; ML, middle leg; HL, hind leg. T2 and T3: mesothorax and metathorax spiracles, respectively. A3: abdominal spiracle (cf. Fig. 6a).

To address the issue of chemical property, we treated specimens using a detergent (see “Methods”), which removes oils or waxes, and hence, can affect the hemispheroidal liquid substances. In the detergent-treated specimens, the wettability of the surface changed from hydrophobic to hydrophilic in the segment of lateral plates (Fig. 8a,d, insets). No hemispheroidal liquid substance was detected on the surface of these specimens (Fig. 7e,f). Moreover, in the treated specimens, when the test solution was applied at site no. 5, higher silica peaks were detected only around the initial application site (*p* < 0.01; Fig. 8; Table 2). In contrasting experiments, the application at no. 6 revealed similar patterns (Supplementary Fig. S2; Table 2). After 3 h, hemispheroidal liquid substances were visible on the body surface even in the detergent-treated specimens, which were still alive after the treatment (Fig. 7g,h). After 6 h, the water contact angles in the lateral plates showed a natural hydrophobicity (as described above) and directional spreading had recovered.

## Discussion

Among the various types of pesticides which are applied as volatiles or as contact solids, we focussed on the liquid type of pesticides in this study. Although aerosol is one of the most popular dosage forms of pesticides, effective extermination occurs when the liquid type of agents adhering to the body surface moves to the points of entry. A specific hydrophobic test solution was prepared that mimics the constitution, viscosity, and adhesiveness of commercial liquid pesticides used for cockroaches (see “Methods”). Non-poisonous component diluted in the common insecticide solvent, n-paraffin, was applied to living specimens in this study. Although the solution was colourless and transparent, as the solution contained silicon oil, silica could be detected at locations where the solution had passed; therefore, silica was the main indicator for the SEM–EDS analysis.

We have previously demonstrated that the combination of the NanoSuit and EDS method facilitates the accurate detection of elemental compositions at a high resolution in living specimens^20^. In the present study, spreading occurred as follows: (i) from the dorsal and/or ventral body surface to the lateral plates (Fig. 3; Supplementary Movie 2; Table 1); ii) from the anterior to the posterior segments along the lateral plates (Fig. 4; Table 2). In this case, spreading in the opposite direction, from the posterior to the anterior segments, was interrupted at a boundary created by two different fine structures (Fig. 5; Table 2). The posterior segments exhibited short, large protrusions, rather densely arranged (Figs. 4g, 6b; compare Figs. 5g, 6d). This effect can be explained by the following hypothesis: the roughness of the structures increases the surface area, which results in hydrophobicity (Wenzel model)^24^. As per this principle, the strength of the capillary force of the hydrophobic solution could be much higher around the hydrophobic dense and short protrusions in the posterior segments. Therefore, although the solution could cross the boundary and spread from the anterior to posterior segments (Fig. 4g), spreading in the opposite direction was interrupted at the boundary (Fig. 5g).

Notably, higher signals were detected in the spiracles if they were localised on the paths of the spreading solution (Fig. 6a,e–g; Table 2). Sugiura et al. reported that the entry of pyrethroids via spiracles is important for knockdown efficacy^14^. They suggested that the knockdown effect is caused by the flow of pesticides into mesothoracic spiracles and its subsequent penetration through the inner wall of the mesothoracic trachea. The present findings further suggest the possibility that the adherence of the pesticide solution to these directional spreading routes is associated with a high probability of pesticides entering spiracles, which could increase insecticidal activity.

Under the high vacuum condition, numerous hemispheroidal liquid substances were observed on the surfaces of living *B. germanica* specimens (Fig. 7a,b). The liquid substances presented high oxygen and low carbon peaks (Fig. 7c,d). Although the specific components of the hemispheroidal liquid substances were not defined in our experiments, these substances could be a mixture of water and the unknown substances (Supplementary Movie 4). These substances were not detected in specimens prepared with conventional fixation methods. This could be because the multistep treatments of samples with various solutions, including ethanol, may result in the removal of components of liquid structures from the sample^15^. Moreover, in specimens treated with the conventional methods, it was impossible to conduct the spreading analyses in the present investigation, because in such specimens, natural surface properties were modified by complex pre-treatments, resulting in poor affinity for the pesticide-mimicking test solutions^15,20^.

Similarly, when the chemical property of the surface was altered with the pre-detergent treatment, no hemispheroidal liquid substances were detected (Fig. 7e,f), and spreading did not occur (Fig. 8; Supplementary Fig. S2; Table 2). However, after 3 h, the liquid substances were visible on the body surface of the detergent-treated specimens (Fig. 7g,h). Overall, results suggest that the liquid substances were originally secreted from inside the living animal and subsequently covered the entire surface^25,26^. The surface had a high affinity for hydrophobic test solutions, and hence, contributed to the spreading. Therefore, further elucidation of the relationship between these chemical properties and pesticides may lead to more effective pesticide use.

As demonstrated above, the morphological features of fine surface structures with short protrusions (Figs. 4, 5) and their chemical characteristics (Figs. 7, 8; Supplementary Fig. S2) regulate the strength of the open capillary force, which determines the mechanism of directional spreading of pesticides (Table 2). Although the detailed functions of the chemically modified fine structures in cockroaches remain unknown, similar morphological structures consisting of belts of scale rows have been observed at a similar position on the sternite in isopods^27,28^. Results from this study suggest that, on the surface of cockroaches, the fine and short protrusions may play a similar role of open capillary force in the transport of the hydrophobic liquid involved in certain physiological regulations. On the natural hydrophobic surface in cockroaches, the adhered hydrophilic solution is not transported on the short protrusions (Supplementary Fig. S3), which supports the present hypothesis.

Although several studies on alternative methods of pest control have been conducted, at present, it is not possible to completely eliminate pesticides. This study demonstrated that specific approaches utilising the natural surface features of pests can facilitate the development of more effective pesticides and control of insect pests by increasing insecticidal activity and decreasing the total volume of reagents used, with reduced toxicity to other animals. Therefore, this approach is an example of eco-mimetics research that aims to solve the dilemma of ensuring ecosystem conservation while achieving increased agricultural production. Finding a method to reduce the quantity of pesticides will significantly contribute toward meeting the Sustainable Development Goals proposed by the United Nations in 2015.

## Methods

### Experimental organisms

Third-instar larvae (~ 6 mm body length) of German cockroaches (*B. germanica* L.) were used in this study. Colonies were obtained from the Institute of Medical Science, University of Tokyo, Japan, and were reared on pelleted feed (NMF; Oriental Yeast Co., Ltd., Tokyo, Japan) in the laboratory of the Research and Development Division, Fumakilla Limited, at room temperature (27 ± 2 °C) under a 14:10 h light:dark (L:D) cycle.

### Microscopy

FE-SEM was performed using a JEM-7100F (JEOL, Tokyo, Japan) and/or a Hitachi S-4800 instrument, operated at an acceleration voltage of 1.0 kV. The vacuum level of the observation chamber was 10^−3^–10^−6^ Pa. The lower detector served as the secondary electron detector. In addition, the working distance was 8 mm, the aperture size *φ* was 100 µm, and the scan speed for each beam was 10–15 frames/s. The beam irradiation density and dose were approximately 2.65 × 10^17^/m^2^ to 9.56 × 10^18^/m^2^, depending on the conditions (for both SEM instruments).

### NanoSuit preparation of live experimental specimens for FE-SEM

The specimens were introduced into the SEM chamber without pre-treatment (for example, chemical fixation, dehydration, or ultrathin coating of electrically conducting materials), where a NanoSuit was then formed following electron beam irradiation (NanoSuit method)^16–18^. A low magnification (20–30×) electron beam was used to irradiate the entire surface of the sample, and the areas in which a NanoSuit formed were used for the subsequent SEM observations^20^.

### Recording movements

To record the dynamic movements of animals in the SEM, imaging data from the SEM were directly transferred to a Hi-band digital formatted video recorder (Pioneer, DVR-DT95) (cf. Supplementary Movie 1). The movements of the test solutions were recorded with a 1000-fps high-speed camera (HAS-220C, DITECT) with a variable lighting system comprising an LED illumination device (LA-HDF158A, HAYASHI-REPIC) (cf. Supplementary Movies 2, 3).

### Energy dispersive X-ray spectrometry

Elemental analysis was performed using a X-Max^n^ (Oxford Instruments, Tokyo, Japan). Two EDS devices were equipped with the SEM, which operated at acceleration voltages of 10.0 kV^20^.

### Reagents in the test solution

The test liquid used in the present study consisted of 5% (wt/v) polydimethylsiloxane (50 mm^2^/s; Shin-Etsu Chemical Co., Tokyo, Japan) dissolved in n-paraffin (Chuo, Kasei Co., Osaka, Japan). In some experiments, 1% (v/v) caesium chloride (Wako Pure Chemical Industries, Osaka, Japan) dissolved in distilled water was used (Supplementary Fig. S3).

### Efficacy tests

Each experimental specimen contained in individual plastic tubes were lightly anesthetised at low temperature (4 °C) for 5 min. The anaesthetised specimens were fixed on the observation stub using double-sided tape for subsequent analysis using SEM (cf. Fig.2e). The test solution (0.2 μL) was applied to six independent sites on the surface of the specimens in each experiment (cf. Fig. 1a) with a glass micropipette (cf. Fig. 1b–d). After 15 min, the treated specimens were placed into the SEM chamber and observed using the NanoSuit method, as described above.

### Quantitative surface hydrophobicity measurement using a contact angle metre

The interfacial tensions arising from the interaction of different surfaces with water were compared in terms of the surface wettability measured using automatic microscopic contact angle metres (Kyowa DMs-401, Japan). A 1 µL droplet of water was formed on the tip of a glass capillary with an inner diameter of 0.5 mm. Water contact angles (*θ*) were measured from the captured images^21^.

### Removing hydrophobic substances from the surface of specimen

In some experiments, prior to adding the test solution at the application sites, the surface of the specimens was treated with the amphiphilic compound, polysorbitan monolaurate (Tween 20; Wako Pure Chemical Industries). The specimens were dipped into 10% (v/v) Tween 20 solution dissolved in distilled water for 10 s, washed three times in distilled water for 10 s, and blotted briefly on dry filter paper to remove excess solution (cf. Fig. 8; Supplementary Fig. S2).

### Statistics and reproducibility

Statistical analysis was carried out using Student’s *t*-test. The tests were two-tailed, and the actual *p* value for each test was generated with the significance level set at *p* < 0.01.

### Supplementary Information

Supplementary Figure S1.

Supplementary Figure S2.

Supplementary Figure S3.

Supplementary Video 1.

Supplementary Video 2.

Supplementary Video 3.

Supplementary Video 4.

Supplementary Legends.

## Data Availability

The authors declare that all data generated or analysed during the study are stored and securely backed-up. In addition, the original datasets including the image sets in the main figures and any remaining information are available from the corresponding authors on reasonable request.
