# Supplementary figures and images for: Route of pesticide spread on the body surface of Blattella germanica (Linnaeus): a NanoSuit–energy dispersive X-ray spectroscopy analysis
Source: Sci Rep. 2023 Aug 31;13:14335. doi: 10.1038/s41598-023-41474-x (PMC10471590; doi:10.1038/s41598-023-41474-x)

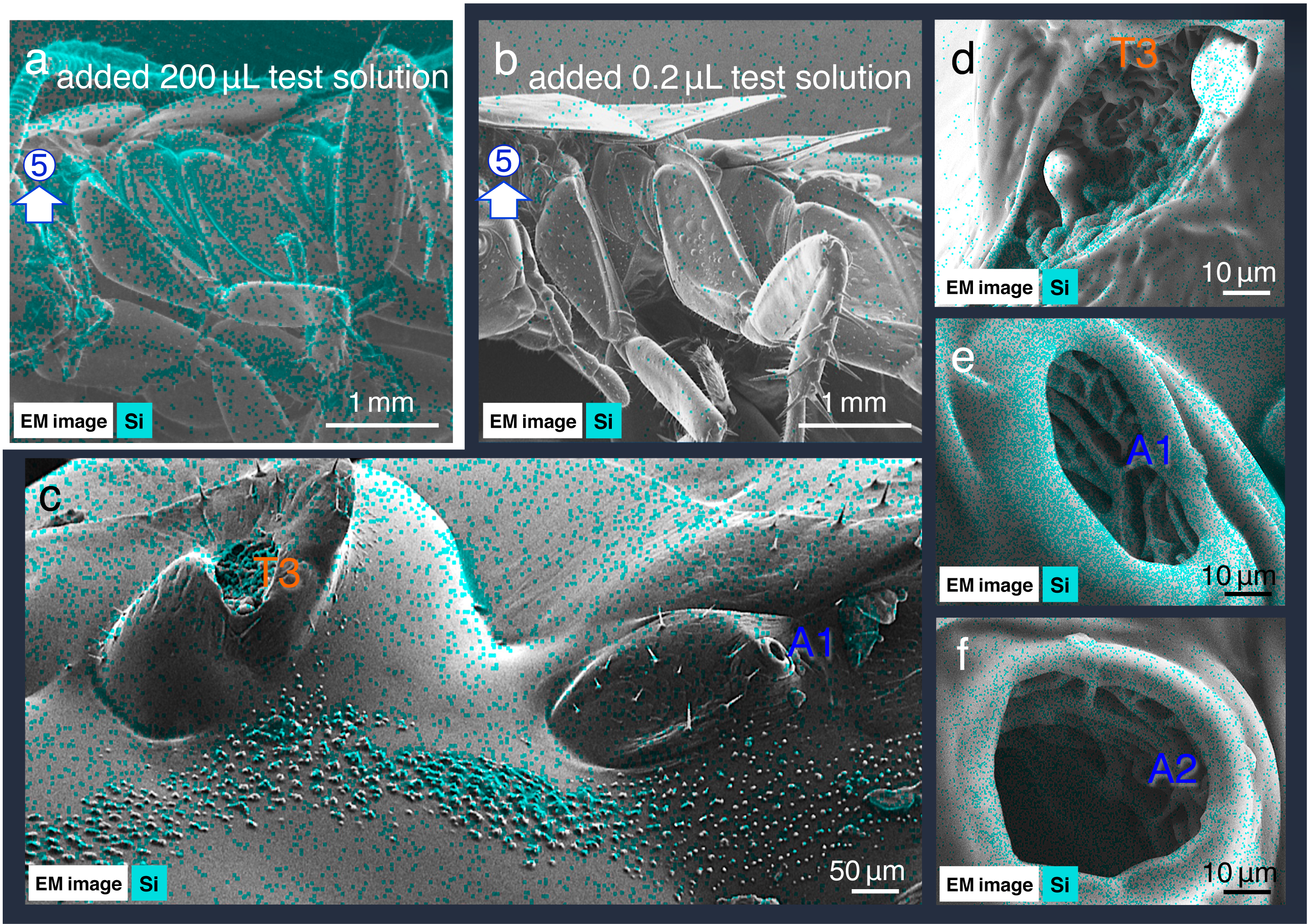

Supplement: Supplementary file 1 — Supplementary Figure S1. [file 41598_2023_41474_MOESM1_ESM.tif]

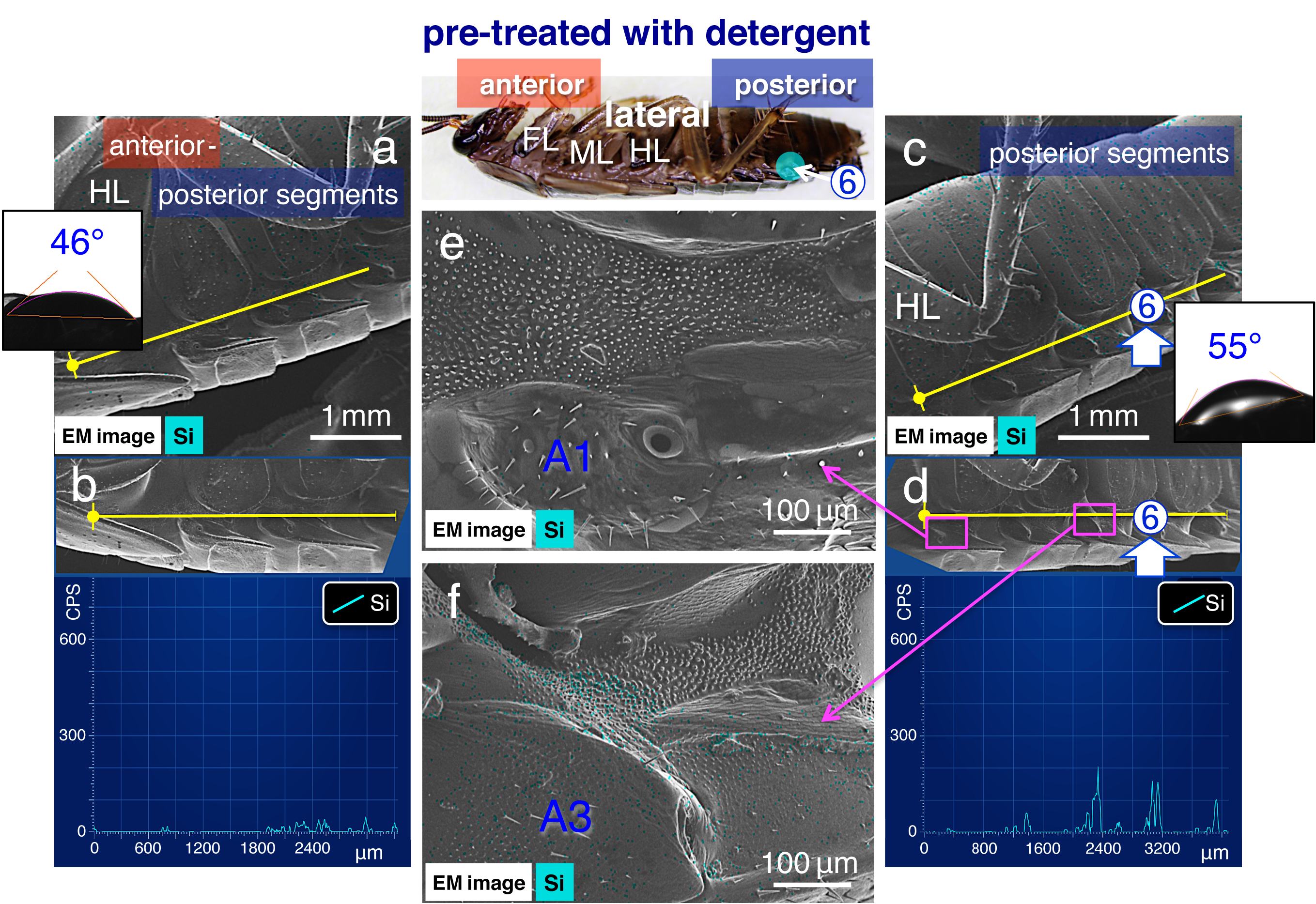

Supplement: Supplementary file 2 — Supplementary Figure S2. [file 41598_2023_41474_MOESM2_ESM.tif]

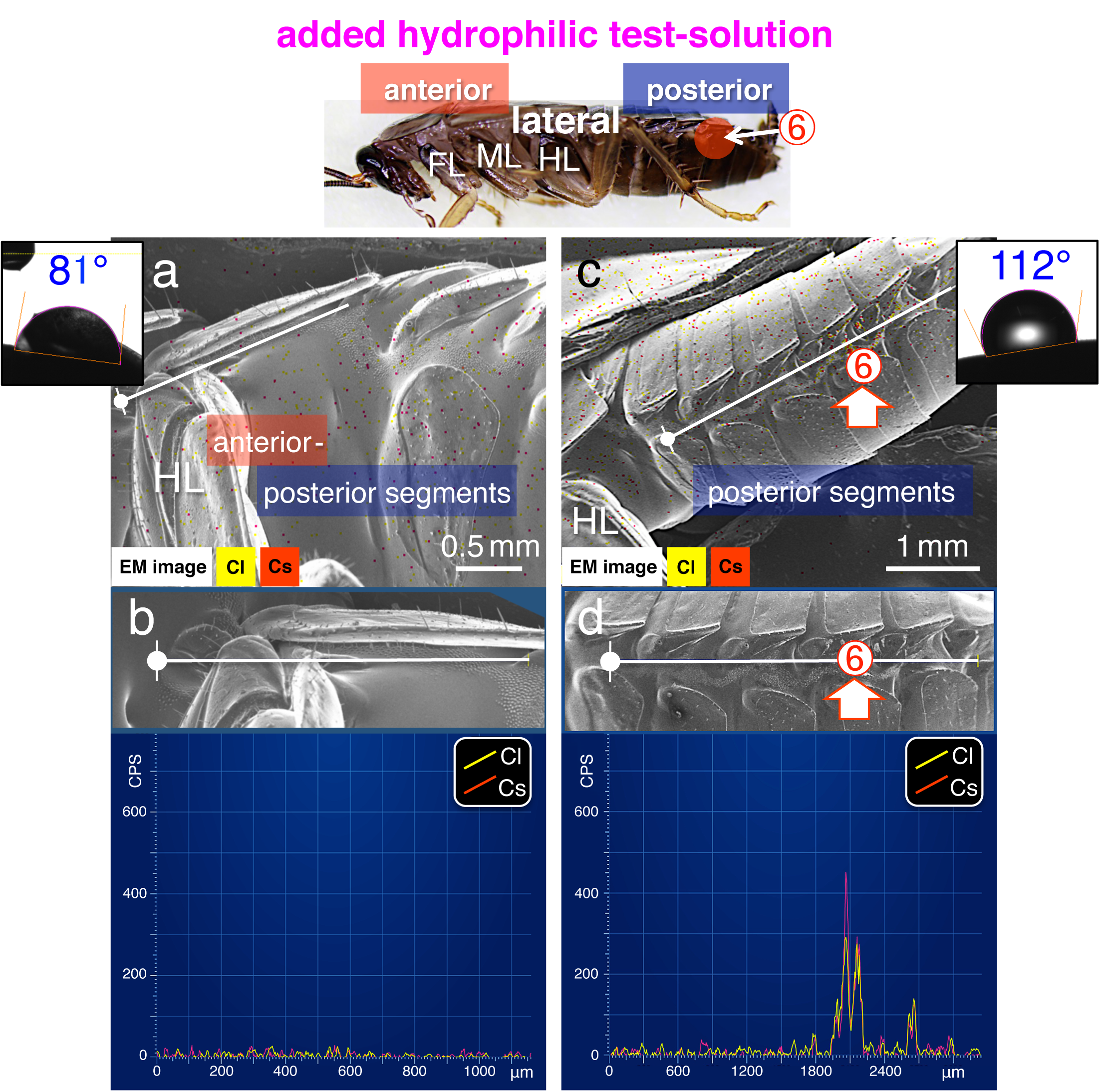

Supplement: Supplementary file 3 — Supplementary Figure S3. [file 41598_2023_41474_MOESM3_ESM.tif]
