# Supplementary material for: Route of pesticide spread on the body surface of Blattella germanica (Linnaeus): a NanoSuit–energy dispersive X-ray spectroscopy analysis
Source: Sci Rep. 2023 Aug 31;13:14335. doi: 10.1038/s41598-023-41474-x (PMC10471590; doi:10.1038/s41598-023-41474-x)
Supplement: Supplementary file 8 — Supplementary Legends. [file 41598_2023_41474_MOESM8_ESM.docx]

**Fig. S1.** Images of the EDS analysis depending on different quantities of the test solution. Images from the EDS “mapping analysis” near application site no. 5 in anterior segments. (**a**) Image of adding a large volume of the test solution (200 μL). (**b–f**) Images of adding a small volume of the test solution (0.2 μL). T3: metathorax spiracle, A1 and A2: abdominal spiracles (cf. Fig. 6a).

**Fig. S2** Images of the EDS analysis of specimens with reduced liquid structures. (**a)** and (**c**) Images from the EDS “mapping analysis” at the end of posterior segments connecting to anterior segments in (**a)** and near application site no. 6 in posterior segments in (**c)** in the detergent-treated specimens. Water contact angle measurements are shown in the insets. (**b)** and (**d**) EDS “line scanning analysis” (along yellow lines) of the same observation field (shown as insets). (**e)** and (**f**) High magnification images of EDS “mapping analysis” near application site no. 6 in (**f)** and in fine structures with short protrusions at the end of the posterior segments connecting to anterior segments in (**e)**. Light blue signal indicates the localisation of elemental silica. FL: front leg, ML: middle leg, and HL: hind leg. A1: abdominal spiracle (cf. Fig. 6a).

**Fig. S3** Images of the EDS analysis conducted using a hydrophilic test solution. (**a)** and **(c)** Image from the EDS “mapping analysis” at the end of posterior segments connecting to anterior segments in (**a)**, and near application site no. 6 in the posterior segments in (**c)**. Water contact angle measurements are shown in the insets. (**b)** and **(d)** EDS “line scanning analysis” (along white lines) of the same observation field (shown as inset). Yellow and red signals indicate the localisation of elemental caesium or chloride, respectively. FL: front leg, ML: middle leg, and HL: hind leg.

**Supplementary Movies—Legends**

**Supplementary Movie 1.** Movements of the living cockroach specimen observed using FE-SEM. The untreated specimen was irradiated with an electron beam (1.0 kV) and exposed to high vacuum (10^-3^–10^-6^ Pa) to observe its fine structure (NanoSuit method).

**Supplementary Movie 2.** Time-lapse top-view video of the ventral surface in posterior segments in the cockroach. The site at which the pesticide-mimicking solution is topically applied (application site No. 4 in Fig. 1a) is shown.

**Supplementary Movie 3.** Time-lapse side-view video of the lateral plate in posterior segments in the cockroach. The site at which the pesticide-mimicking solution is topically applied (application site No. 6 in Fig. 1a) is shown.

**Supplementary Movie 4.** Active conformational changes of hemispheroidal-shaped liquid substances (cf. Fig. 7). When the magnification increased 1000× to 5000×, the liquid substances evaporated within 30 s.
